# Complete genome sequence of *Paenibacillus yonginensis* DCY84^T^, a novel plant Symbiont that promotes growth via induced systemic resistance

**DOI:** 10.1186/s40793-017-0277-8

**Published:** 2017-10-13

**Authors:** Yeon-Ju Kim, Johan Sukweenadhi, Ji Woong Seok, Chang Ho Kang, Eul-Su Choi, Sathiyamoorthy Subramaniyam, Deok Chun Yang

**Affiliations:** 1 Graduate School of Biotechnology, College of Life Science, Kyung Hee University, Yongin, 446-701 South Korea; 2 Lab Genomics Co. Ltd, Jinju, South Korea; 3 Division of Applied Life Science and PMBBRC, Gyeongsang National University, Jinju, South Korea

**Keywords:** *Paenibacillus yonginensis* DCY84^T^, Genome, PacBio, Plant growth promoting rhizobacteria (PGPR)

## Abstract

This article reports the full genome sequence of *Paenibacillus yonginensis* DCY84^T^ (KCTC33428, JCM19885), which is a Gram-positive rod-shaped bacterium isolated from humus soil of Yongin Forest in Gyeonggi Province, South Korea. The genome sequence of strain DCY84^T^ provides greater understanding of the *Paenibacillus* species for practical use. This bacterium displays plant growth promotion via induced systemic resistance of abiotic stresses.

**Electronic supplementary material:**

The online version of this article (10.1186/s40793-017-0277-8) contains supplementary material, which is available to authorized users.

## Introduction

Various 10.1601/nm.5109 species constitute a large group of facultative anaerobic endospore-forming Gram-positive bacteria that are extensively distributed in nature. Ash et al. proposed that members of ‘group 3’ within the genus 10.1601/nm.4857 should be transferred to the genus 10.1601/nm.5109, for which they proposed 10.1601/nm.5110 as the type species [1] Since that time, 174 different type species have been described.

Members of the genus 10.1601/nm.5109 are well known as PGPR, together with 10.1601/nm.2713 *,* 10.1601/nm.822 *,* 10.1601/nm.2552 *,* 10.1601/nm.857 *,* and 10.1601/nm.1619 [2]. While many new species from the genus 10.1601/nm.5109 have been reported [3], the type species 10.1601/nm.5110 [4] is considered a PGPR that is widely used in sustainable agriculture and environmental remediation because of its multiple functions [2, 5]. Coupled with many plant species, some 10.1601/nm.5109 species have been developed as biofertilizers or biocontrol agents and have been used effectively in the control of plant-pathogenic fungi, bacteria, and nematodes [5–7]. 10.1601/nm.26227 DCY84^T^ was isolated from a decomposed humus mixture in South Korea and its plant growth promotion traits have been characterized in vitro [8]. This strain is capable of inducing the defense response of *Arabidopsis* against several abiotic stresses [9]. Genome sequencing of *P. yonginensis* DCY84^T^ was conducted to obtain additional insights into the physiological characteristics involved in microbe-plant interactions and to facilitate better understanding of the molecular basis of these traits.

## Organism information

### Classification and features

**Fig. 1:**
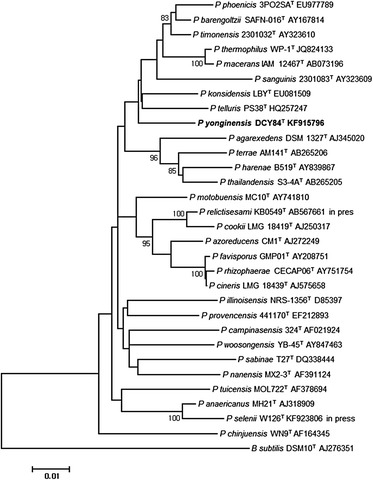
Phylogenetic tree highlighting the position of *Paenibacillus yonginensis* DCY84^T^ relative to other *Paenibacillaceae* family type strains. GenBank accession numbers are indicated in parentheses. Sequences were aligned using CLUSTAL X (V2), and phylogenetic inferences were obtained using the maximum-likelihood method

**Fig. 2:**
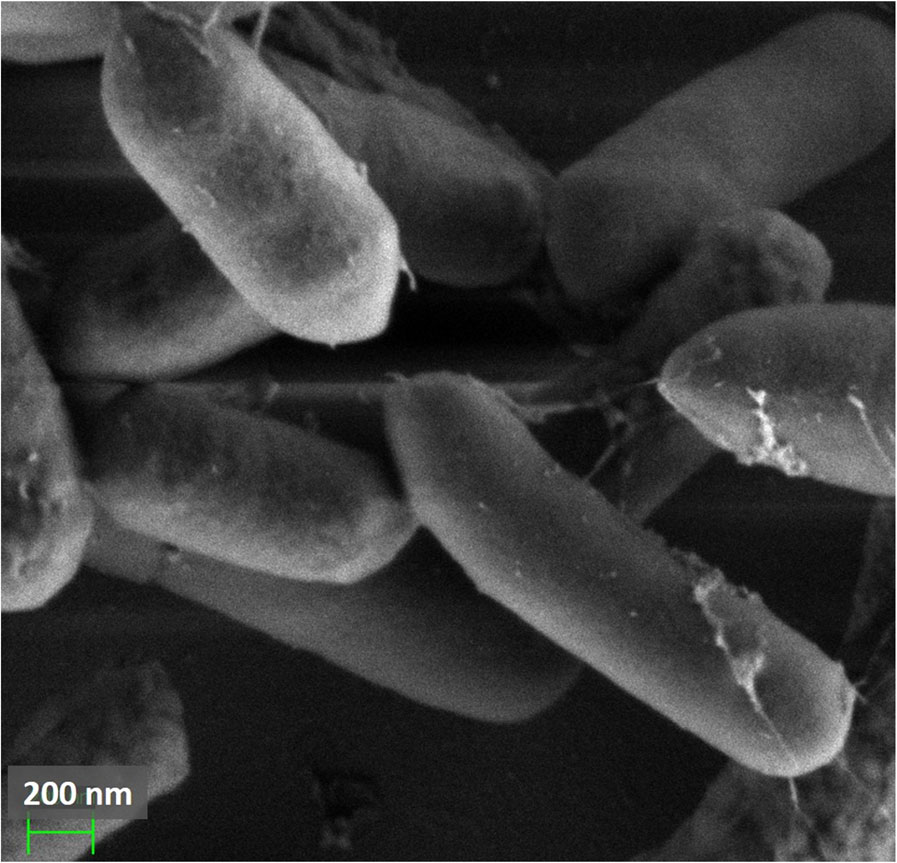
Scanning electron microscopy image of strain DCY84^T^

10.1601/nm.26227 DCY84^T^ was isolated from a decomposed humus mixture collected from Yongin province. It is a Gram-positive bacterium that can grow on Tryptic soy broth agar at 28 °C. Cells of strain DCY84^T^ are rod-shaped with a diameter ranging from 0.7–0.9 μm and length ranging from 3.4 to 4.7 μm. Growth occurs under aerobic conditions with an optimum growth temperature at 25–30 °C and a temperature range of 15–40 °C, general features of strain DCY84^T^ were presented in Table 1. Phylogenetic tree highlighting the position of 10.1601/nm.26227 DCY84^T^ and phylogenetic inferences were obtained using the maximum-likelihood method (Fig. 1). Cell morphology was examined using scanning electron microscopy (Fig. 2).

**Table 1:** Classification and general features of *Paenibacillus yonginensis* DCY84^T^

MIGS ID	Property	Term	Evidence Code
	Classification	Domain Bacteria	TAS [17]
		Phylum *Firmicutes*	TAS [18, 19]
		Class *Bacilli*	TAS [20]
		Order *Bacillales*	TAS [21, 22]
		Family *Paenibacillaceae*	TAS [21, 23]
		Genus *Paenibacillus*	TAS [15]
		Species *Paenibacillus yonginensis*	TAS [8, 9]
		Strain DCY84^T^	TAS [8, 9]
	Gram stain	positive	IDA
	Cell shape	rod	IDA
	Motility	motile	IDA
	Sporulation	spore production	IDA
	Temperature range	15–40 °C	IDA
	Optimum temperature	30 °C	IDA
	pH range; Optimum	5–9; 8	IDA
	Carbon source	D-Xylose, D-ribose, D-glucose and others	TAS [8]
MIGS-6	Habitat	humus soil	IDA
MIGS-6.3	Salinity	0.5–4.5% NaCl	IDA
MIGS-22	Oxygen requirement	Aerobic	IDA
	Carbon source	glucose, lactose	TAS [8]
MIGS-15	Biotic relationship	Free-living	IDA
MIGS-14	Pathogenicity	Non-pathogenic	NAS
MIGS-13	Source material identifiers	KCTC 33428^T^, JCM 19885^T^	TAS [8]
MIGS-4	Geographic location	South Korea: Gyeonggi province	IDA
MIGS-5	Sample collection	September 2013	IDA
MIGS-4.1	Latitude	37.314 N	IDA
MIGS-4.2	Longitude	127.268 W	IDA
MIGS-4.4	Altitude	131.37 m	IDA

Evidence codes: *IDA* inferred from direct assay, *TAS* traceable author statement (i.e., a direct report exists in the literature), and *NAS* non-traceable author statement (i.e., not directly observed for the living, isolated sample, but based on a generally accepted property for the species, or anecdotal evidence). These evidence codes are from the Gene Ontology project [24]

## Genome sequencing information

### Genome project history

10.1601/nm.26227 DCY84^T^ was selected for genome sequencing because we observed the presence of a unique compatible solute for plant protection from biotic stress and potential plant growth promoting activity with rice in reclaimed paddy soil and *Panax ginseng* C.A.Mey, respectively. The complete genome sequence has been deposited in the NCBI sequencing read archive under NCBI BioProject PRJNA306396 with BioSample SAMN04419545 and overall sequencing project information was presented in Table 2. Sequencing, annotation, and analysis were performed at LabGenomics (Seongnam, Republic of Korea).

**Table 2:** Genome sequencing project information for *Paenibacillus yonginensis* DCY84^T^

MIGS ID	Property	Term
MIGS 31	Finishing quality	Finished
MIGS-28	Libraries used	Pacbio SMRTbell™ library
MIGS 29	Sequencing platforms	PacBio RS
MIGS 31.2	Fold coverage	130X
MIGS 30	Assemblers	SMRT Analysis v2.3.0 HGAP.2
MIGS 32	Gene calling method	Glimmer 3.02 ex: Prodigal, GenePRIMP
	Locus Tag	AWM70
	GenBank ID	CP014167
	GenBank Date of Release	January 28, 2016
	GOLD ID	Gp0177323
	BIOPROJECT	PRJNA306396
MIGS 13	Source Material Identifier	KCTC 33428^T^, JCM 19885^T^
	Project relevance	Taxonomy, agriculture, plant–microbe interactions

### Growth conditions and genomic DNA preparation

For growth and genomic DNA preparation, 10.1601/nm.26227 DCY84^T^ (10.1601/strainfinder?urlappend=%3Fid%3DKCTC+33428 ^T^=10.1601/strainfinder?urlappend=%3Fid%3DJCM+19885 ^T^) was grown in DSMZ medium 1 (Nutrient Agar) at 28 °C. DNA was isolated from 0.5–1 g of cell paste using the JetFlex genomic protocol as recommended by the manufacturer. For genome sequencing and assembly, the draft genome of 10.1601/nm.26227 DCY84^T^ was generated using the PacBio platform following the manufacturer’s instructions.

### Genome sequencing and assembly

Sequencing produced 74,264 reads with an average length of 7828 bp, which was assembled using *the* de novo HGAP implemented within the analysis pipeline SMRT Analysis 2.2 (Pacific Biosciences, CA, USA). Ambiguous base and inserted/deleted regions between the PacBio assembled and preassembled high quality draft sequences were manually corrected using consensus sequences for final assembly. Long reads were selected as the seed sequences for constructing preassemblies, and the other short reads were mapped to the seeds using BLASTR software for alignment, which corrected errors in the long reads and thus increased the accuracy rating of bases. The sequencing run yielded 581,398,217 filtered and sub-read bases and a total of 113,985,693 pre-assembled bases were used for deep sequencing. tRNA and rRNA genes were identified by tRNAscan-SE version 1.3 [10] and RNAmmer version 1.2 [11]. The ORFs were predicted using Glimmer 3.02 and the annotation of predicted genes was conducted using Blastall 2.2.26. Protein coding genes were annotated based on the COGs database.

### Genome annotation

**Fig. 3:**
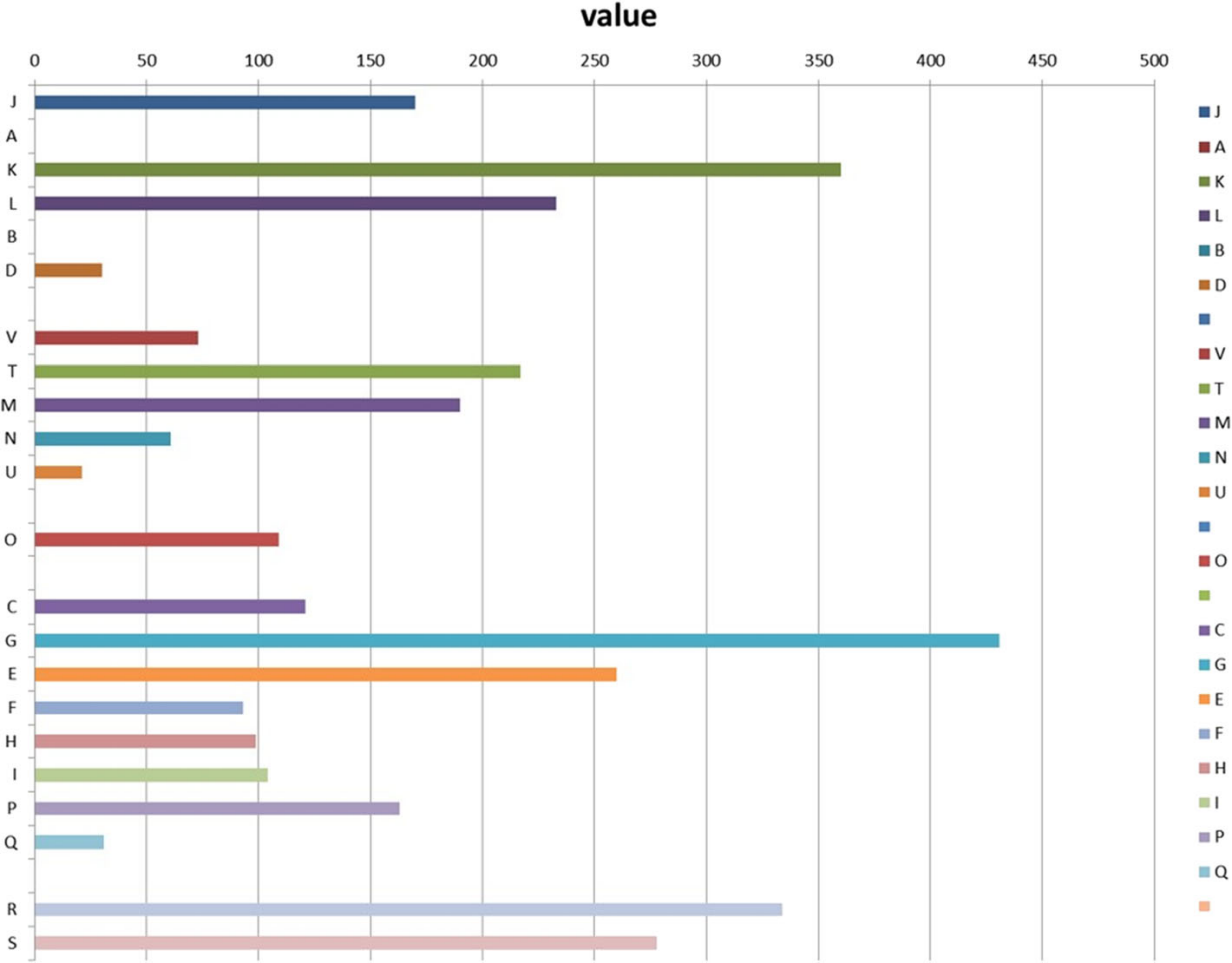
COG analysis of strain DCY84^T^

The purpose of the present study was to develop a better understanding of the 10.1601/nm.26227 DCY84^T^ genetic background to develop more effective utilization of the strain. COGs analysis of strain DCY84^T^ is shown in Fig. 3 and the number of genes associated with the 22 general COGs functional categories presented in Table 3. The analysis of the full 10.1601/nm.26227 DCY84^T^ genome in comparison with other related 10.1601/nm.5109 strains is included in Additional file 1: Table S1.

**Table 3:** Number of genes associated with the 22 general COG functional categories

Code	Value	%age^a^	Description
J	170	4.02	Translation, ribosomal structure and biogenesis
A	0	0.00	RNA processing and modification
K	360	8.50	Transcription
L	233	5.50	Replication, recombination and repair
B	0	0.00	Chromatin structure and dynamics
D	30	0.71	Cell cycle control, cell division, chromosome partitioning
V	73	1.72	Defense mechanisms
T	217	5.13	Signal transduction mechanisms
M	190	4.49	Cell wall/membrane/envelope biogenesis
N	61	1.44	Cell motility
U	21	0.50	Intracellular trafficking, secretion, and vesicular transport
O	109	2.58	Posttranslational modification, protein turnover, chaperones
C	121	2.86	Energy production and conversion
G	431	10.18	Carbohydrate transport and metabolism
E	260	6.14	Amino acid transport and metabolism
F	93	2.20	Nucleotide transport and metabolism
H	99	2.34	Coenzyme transport and metabolism
I	104	2.46	Lipid transport and metabolism
P	163	3.85	Inorganic ion transport and metabolism
Q	31	0.73	Secondary metabolites biosynthesis, transport and catabolism
R	334	7.89	General function prediction only
S	278	6.57	Function unknown
–	1372	32.41	Not in COGs
^b^Total	4750	112.21	

^a^The percentage is based on the total number of protein coding genes in the annotated genome

^b^The total does not correspond to 4498 CDS because some genes are associated with more than one COG functional categories

The *iaaM* gene, also gene responsible for IAA synthesis, siderophores production, phosphate transporter, phosphonate cluster, antimicrobial production, and synthesis of the volatile organic compound *bdhA* are present in the 10.1601/nm.26227 DCY84^T^ genome. These genes corroborate with our physiological results demonstrating plant growth promotion and induced systemic resistance in the plant symbiont [9, 10].

## Insights from the genome sequence

**Fig. 4:**
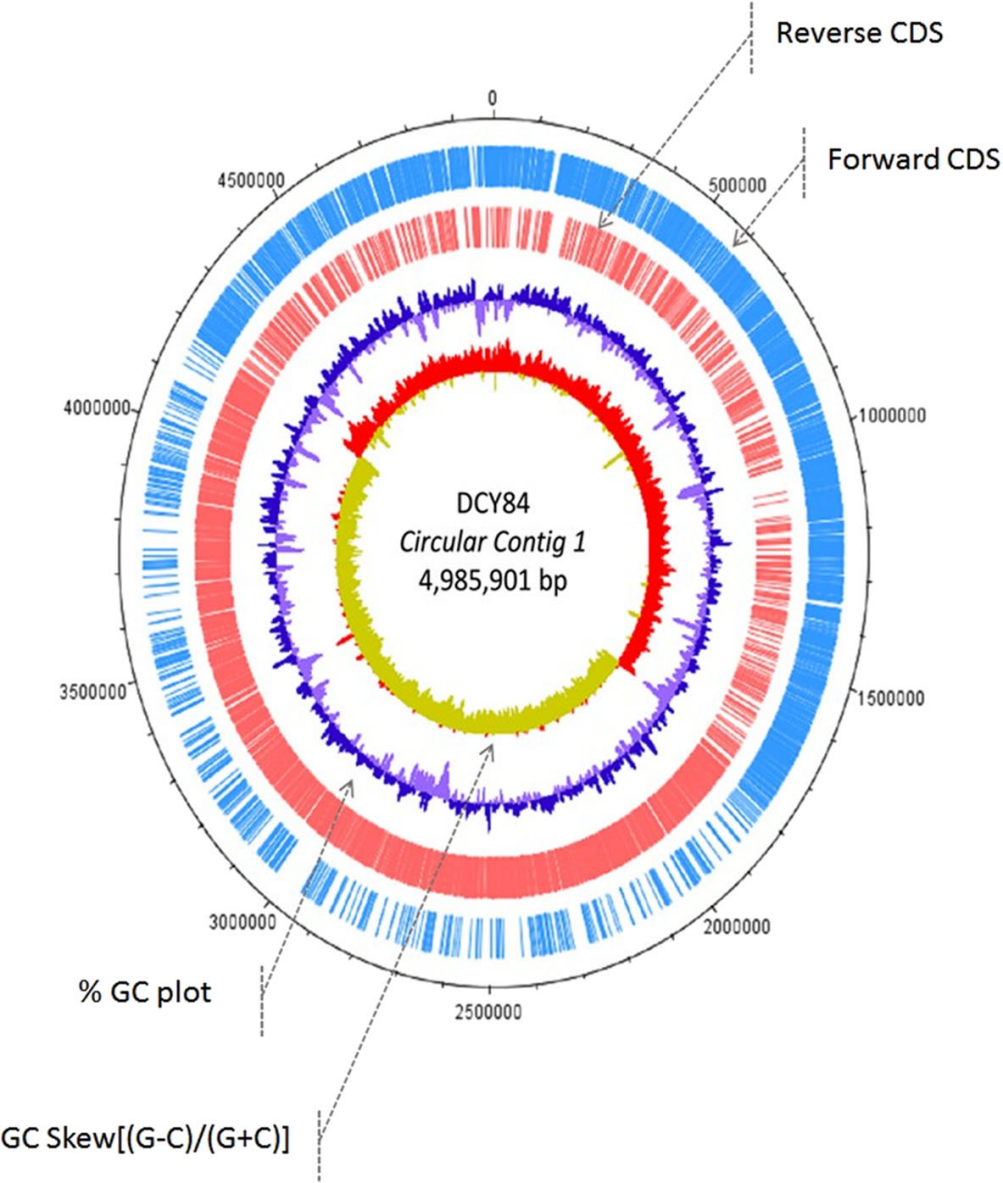
Graphical circular map of the chromosome. From the outside to the center, genes on the forward strand are colored by COG categories (only genes assigned to COG), genes on the reverse strand are colored by COG categories (only genes assigned to COG), RNA genes (tRNAs green, rRNAs red), G + C content, and GC skew. Purple and olive colors indicate negative and positive values, respectively

The completed 10.1601/nm.26227 DCY84^T^ genome consists of a single circular chromosome of 4,985,901 bp, with a GC content of 51.01%, which is similar to most 10.1601/nm.5109 strains (45 – 54%) as reported previously [12] (Fig. 4). The genome size of the strain DCY84^T^ (4.985 Mb) is smaller than the other sequenced members of genus 10.1601/nm.5109 including 10.1601/nm.5110 CF05 (5.76 Mb), and 10.1601/nm.15028 3016 (8.74 Mb) [13]. Full genome of DCY84^T^ was annotated by following NCBI prokaryotic genome annotation pipeline [14]. A total of 4498 genes were predicted for the genome, including 4233 coding sequences (94.1% of total genes) and 147 pseudo genes. Nucleotide content and gene count levels of the chromosome were summarized in Table 4. More detail annotation of the strain DCY84^T^ was available in Additional file 2: Table S5. Most of selected 10.1601/nm.5109 strain was reported to have plant growth promoting factor traits. The summary features of DCY84^T^ and referred strains are showed on Additional file 1: Table S1 below, including the genome accession number, genome size, GC content, annotation information, protein, Gene, Pseudo gene. The COGs analysis of strain DCY84^T^ and other closely related 10.1601/nm.5109 strains was provided on Additional file 1: Table S2 (direct plant growth promoting factors) and Additional file 1: Table S3 (indirect plant growth promoting factors). The genome of 10.1601/nm.26227 DCY84^T^ and 10.1601/nm.5110 M1 were visualized in Additional file 3: Figure S1 by the comparison using the Artemis software and ACT [15]. Strain DCY84^T^ increased nutrient availability by producing several hydrolyzing enzymes, amino acid transporter proteins (Additional file 1: Table S4). Moreover, Strain DCY84^T^ treatment can induce plant defense mechanism mediated by ABA signal under salinity stress.

**Table 4:** Genome statistics

Attribute	Value	% of Total
Genome size (bp)	4,985,901	100.0
DNA coding (bp)	4,267,050	85.6
DNA G + C (bp)	2,543,529	51.0
DNA scaffolds	1	100.0
Total genes	4498	100.0
Protein coding genes	4233	94.1
RNA genes	118	2.6
Pseudo genes	121	2.7
Genes in internal clusters	792	17.6
Genes with function prediction	4380	97.4
Genes assigned to COGs	3378	75.1
Genes with Pfam domains	2661	59.2
Genes with signal peptides	295	6.6
Genes with transmembrane helices	1197	26.6
CRIPSR repeats	4	–

### Extended insights

Genome analysis showed that 10.1601/nm.26227 DCY84^T^ contained many genes related to the stress response, such as IAA, choline, glutamate decarboxylase and malate transporters, potassium uptake protein, heat shock proteins, chaperone proteins, and sugar transporters. These genes most likely allow the strain to cope with different environmental stresses. Experimentation and additional analysis of these genes may help to elucidate the mechanisms mediating the stress response and facilitate the development of 10.1601/nm.26227 DCY84^T^ as a biofertilizer. When the strain DCY84^T^ was used as a treatment for early sprouting rice seeds, several genes responsible for primary metabolism were upregulated in the rice root, which could be related to PGPR. These results indicate that 10.1601/nm.26227 DCY84^T^ might have the potential for application in industrial biotechnology as a producer of miscellaneous hydrolases.

This is the first report describing the genome sequence of 10.1601/nm.26227 DCY84^T^. When coated on sprouting rice seeds or seedlings directly on paddy soil, strain DCY84^T^ and silica zeolite complex were shown to enhance rice yield and also increase GABA content in brown rice. Treatment was also shown to induce systemic stress resistance responses in rice and *Arabidopsis* under heavy metal and salty conditions. Furthermore, the sequence of 10.1601/nm.26227 DCY84^T^ provides useful information and may contribute to agricultural applications of 10.1601/nm.5109 genera in practical biotechnology. Rice yield was affected by the amount of strain DCY84^T^ administered during the early sprouting stage. Silica zeolite complex and strain DCY84^T^ treatment inhibited the occurrence of fungal infection, and also enhanced rice quality. Silica zeolite complex and two treatments with strain DCY84^T^ resulted in the highest head rice levels (86.8%) compared to a one-time treatment of DCY84^T^ (67.9%), and without strain DCY84^T^ treatment (46.4%). The PGPR treatment enhanced head rice levels by 40.4% [16]. Strain treatment also enhanced nitrogen uptake and increased levels of stored nitrogen in the rice grain, indicating that the strain DCY84^T^ enhanced plant nitrogen utilization with less nitrogen fertilizer application. The most important parameters for economic rice value are head rice rate and good appearance; strain DCY84^T^ treatment enhanced both the rice quality and reduced commercial nitrogen fertilizer usage.

## Conclusion

The DCY84^T^ strain was isolated from a decomposed humus mixture. Phylogenetic analysis based on the 16S rRNA gene confirmed its affiliation to the genus 10.1601/nm.5109. G + C content, COGs, and average nucleotide identities are presented. The genomic features of strain DCY84^T^ are consistent with the plant growth promoting activity of this strain, including IAA production, phosphate solubilizing activity, and siderophores production. In addition, DCY84^T^ induced systemic stress resistance mechanisms in rice and *Arabidopsis* under heavy metal and salty conditions.

## Additional files

Additional file 1: Table S1. Genome comparison of strain DCY84^T^ and closest Paenibacillus strains. **Table S2.** COGs analysis of direct plant growth promoting traits. **Table S3.** COGs analysis of indirect plant growth promoting traits. **Table S4.** Some important genes annotated on strain DCY84^T^ genome. (DOCX 81 kb)

Additional file 2: Table S5. Annotation of the *Paenibacillus yonginensis* DCY84^T^ genome. (XLSX 443 kb)

Additional file 3: Figure S1. Comparative genome analysis of *P. yonginensis* DCY84^T^ and *P. polymyxa* M1 using the Artemis software and ACT. (TIFF 16717 kb)

## Abbreviations

*bdhA*: 2,3-butanediol synthesis
COGs: Clusters of Orthologous Groups of proteins
HGAP: Hierarchical Genome Assembly Process
IAA: Indole-3-acetic acid
*iaaM*: Tryptophan monooxygenase
ORFs: Open Reading Frames
PGPR: Plant Growth Promoting Rhizobacteria
SMRT: Single Molecule, Real-Time
