# Supplementary material for: Complete genome sequence of Paenibacillus yonginensis DCY84T, a novel plant Symbiont that promotes growth via induced systemic resistance
Source: Stand Genomic Sci. 2017 Oct 13;12:63. doi: 10.1186/s40793-017-0277-8 (PMC5640943; doi:10.1186/s40793-017-0277-8)
Supplement: Supplementary file 1 — Genome comparison of strain DCY84T and closest Paenibacillus strains. Table S2. COGs analysis of direct plant growth promoting traits. Table S3. COGs analysis of indirect plant growth promoting traits. Table S4. Some important genes annotated on strain DCY84T genome. (DOCX 81 kb) [file 40793_2017_277_MOESM1_ESM.docx]

**Table S1.** Genome comparison of strain DCY84^T^ and closest *Paenibacillus* strains

| **Strain Name** | **Max Score** | **Total Score** | **Query Cover** | **Ident** | **RefSeq** | **Size (Mb)** | **GC%** | **Protein** | **Gene** | **Pseudo gene** |
| --- | --- | --- | --- | --- | --- | --- | --- | --- | --- | --- |
| *Paenibacillus polymyxa* M1 | 5382 | 1.14E+06 | 4 % | 84 % | NC_017542.1 | 5.86 | 45.2 | 5,067 | 5,279 | 59 |
| *Paenibacillus stellifer* DSM 14472 | 4854 | 1.14E+06 | 8 % | 82 % | NZ_CP009286.1 | 5.66 | 53.5 | 4,899 | 5,167 | 147 |
| *Paenibacillus polymyxa* Sb3-1 | 5376 | 1.13E+06 | 4 % | 84 % | NZ_CP010268.1 | 5.6 | 45.5 | 4,636 | 4,870 | 82 |
| *Paenibacillus polymyxa* CF05 | 5363 | 1.13E+06 | 4 % | 87 % | NZ_CP009909.1 | 5.76 | 45.5 | 4,715 | 4,928 | 63 |
| *Paenibacillus polymyxa* SC2 | 5382 | 1.10E+06 | 4 % | 84 % | NC_014622.2 | 5.73 | 45.2 | 4,897 | 5,107 | 59 |
| *Paenibacillus peoriae* HS311 | 5461 | 1.07E+06 | 4 % | 84 % | NZ_CP011512.1 | 6.01 | 45.7 | 4,963 | 5,182 | 80 |
| *Paenibacillus polymyxa* SQR-21 | 5363 | 1.07E+06 | 4 % | 87 % | NZ_CP006872.1 | 5.83 | 45.6 | 4,768 | 5,030 | 111 |
| *Paenibacillus mucilaginosus* 3016 | 4065 | 1.06E+06 | 4 % | 92 % | NC_016935.1 | 8.74 | 58.3 | 6,700 | 7,324 | 408 |
| *Paenibacillus riograndensis* SBR5 | 4935 | 1.05E+06 | 9 % | 83 % | NZ_LN831776.1 | 7.92 | 51 | 6,467 | 6,691 | 109 |
| *Paenibacillus* sp. FSL P4-0081 | 8990 | 1.04E+06 | 8 % | 80 % | NZ_CP009280.1 | 8.42 | 51.1 | 6,916 | 7,190 | 158 |
| *Paenibacillus mucilaginosus* K02 | 4084 | 1.01E+06 | 4 % | 92 % | NC_017672.3 | 8.82 | 58.3 | 7,016 | 7,289 | 83 |
| *Paenibacillus mucilaginosus* KNP414 | 4060 | 9.91E+05 | 4 % | 92 % | NC_015690.1 | 8.66 | 58.4 | 6,919 | 7,179 | 105 |
| *Paenibacillus polymyxa* E681 | 5461 | 9.87E+05 | 4 % | 84 % | NC_014483.2 | 5.39 | 45.8 | 4,528 | 4,796 | 140 |
| *Paenibacillus polymyxa* CR1 | 5439 | 9.82E+05 | 4 % | 84 % | NC_023037.2 | 6.02 | 45.6 | 5,029 | 5,301 | 150 |
| *Paenibacillus* *graminis* DSM 15220 | 5046 | 9.80E+05 | 8 % | 83 % | NZ_CP009287.1 | 7.17 | 50.6 | 5,982 | 6,208 | 107 |
| *Paenibacillus* sp. JDR-2 | 6423 | 9.57E+05 | 5 % | 78 % | NC_012914.1 | 7.18 | 50.3 | 6,128 | 6,322 | 69 |
| *Paenibacillus* sp. FSL R5-0912 | 8968 | 9.53E+05 | 8 % | 80 % | NZ_CP009282.1 | 7.72 | 51.1 | 6,344 | 6,580 | 117 |
| *Paenibacillus* sp. FSL R7-0273 | 5118 | 9.45E+05 | 8 % | 83 % | NZ_CP009283.1 | 7.29 | 51.9 | 6,051 | 6,259 | 93 |
| *Paenibacillus* sp. IHBB 10380 | 6885 | 9.43E+05 | 3 % | 92 % | NZ_CP010976.1 | 5.77 | 41.3 | 4,950 | 5,217 | 142 |
| *Paenibacillus borealis* DSM 13188 | 9191 | 9.36E+05 | 7 % | 80 % | NZ_CP009285.1 | 8.16 | 51.4 | 6,698 | 6,945 | 130 |
| *Paenibacillus* sp. FSL R7-0331 | 5077 | 9.29E+05 | 7 % | 83 % | NZ_CP009284.1 | 6.93 | 51.2 | 5,815 | 6,028 | 97 |
| *Paenibacillus sabinae* T27 | 5005 | 8.91E+05 | 8 % | 83 % | NZ_CP004078.1 | 5.27 | 52.6 | 4,634 | 4,857 | 114 |
| *Paenibacillus* sp. Y412MC10 | 7528 | 8.63E+05 | 8 % | 84 % | NC_013406.1 | 7.12 | 51.2 | 6,152 | 6,302 | 51 |

**Table S1.** (continued)

| **Strain Name** | **Max Score** | **Total Score** | **Query Cover** | **Ident** | **RefSeq** | **Size (Mb)** | **GC%** | **Protein** | **Gene** | **Pseudo gene** |
| --- | --- | --- | --- | --- | --- | --- | --- | --- | --- | --- |
| *Paenibacillus durus* DSM 1735 | 4944 | 8.58E+05 | 7 % | 82 % | NZ_CP009288.1 | 6.04 | 50.8 | 5,114 | 5,356 | 130 |
| *Paenibacillus* sp. FSL R5-0345 | 5073 | 8.44E+05 | 4 % | 83 % | NZ_CP009281.1 | 6.79 | 43.7 | 5,781 | 5,993 | 92 |
| *Paenibacillus* sp. FSL H7-0737 | 5007 | 8.31E+05 | 8 % | 80 % | NZ_CP009279.1 | 6.77 | 43.7 | 5,711 | 5,904 | 71 |
| *Paenibacillus* sp. FSL H7-0737 | 5007 | 8.31E+05 | 4 % | 83 % | NZ_CP009279.1 | 6.77 | 43.7 | 5,711 | 5,904 | 71 |
| *Brevibacillus* *brevis* NBRC 100599 | 3295 | 8.23E+05 | 1 % | 86 % | NC_012491.1 | 6.3 | 47.3 | 5,690 | 5,929 | 66 |
| *Paenibacillus* *odorifer* DSM 15391 | 5092 | 8.19E+05 | 3 % | 83 % | NZ_CP009428.1 | 6.81 | 44.2 | 5,752 | 5,945 | 76 |
| *Paenibacillus terrae* HPL-003 | 5385 | 8.09E+05 | 5 % | 84 % | NC_016641.1 | 6.08 | 46.8 | 5,137 | 5,396 | 141 |
| *Paenibacillus beijingensis* DSM 24997 | 6549 | 7.39E+05 | 4 % | 78 % | NZ_CP011058.1 | 5.75 | 52.5 | 4,930 | 5,213 | 165 |
| *Brevibacillus* *laterosporus* LMG 15441 | 3260 | 6.42E+05 | 1 % | 86 % | NZ_CP007806.1 | 5.11 | 41.1 | 4,268 | 4,495 | 78 |
| *Thermobacillus* *composti* KWC4 | 3880 | 5.93E+05 | 4 % | 90 % | NC_019897.1 | 4.21 | 60.6 | 3,625 | 3,878 | 151 |
| *Paenibacillus* *larvae* subsp. *larvae* DSM 25430 | 3768 | 5.82E+05 | 2 % | 91 % | NC_023134.1 | 4.05 | 45 | 3,671 | 3,981 | 203 |
| *Bacillus* *subtilis* 168 | 3101 | 5.09E+05 | 1 % | 86 % | NC_000964.3 | 4.22 | 43.5 | 4,175 | 4,421 | 68 |
| *Bacillus coagulans* 36D1 | 3020 | 4.96E+05 | 1 % | 85 % | NC_016023.1 | 3.55 | 46.5 | 3,243 | 3,430 | 73 |
| *Bacillus* *halodurans* C-125 | 3238 | 4.36E+05 | 1 % | 87 % | NC_002570.2 | 4.2 | 43.7 | 3,903 | 4,076 | 68 |
| *Bacillus* *pseudofirmus* OF4 | 3332 | 3.79E+05 | 1 % | 87 % | NC_013791.2 | 3.86 | 40.3 | 3,704 | 3,841 | 40 |
| *Bacillus coagulans* HM-08 | 5443 | 3.63E+05 | 1 % | 86 % | NZ_CP010525.1 | 3.62 | 46.3 | 3,335 | 3,551 | 110 |
| *Clostridium kluyveri* DSM 555 | 8970 | 2.45E+05 | 1 % | 95 % | NC_009706.1 | 3.96 | 32 | 3,712 | 3,824 | 31 |
| *Mahella australiensis* 50-1 BON | 5710 | 1.08E+05 | 1 % | 86 % | NC_015520.1 | 3.14 | 43.5 | 2,853 | 2,950 | 39 |
| *Agrobacterium* *fabrum* C58 | 1103 | 4.90E+04 | 0 % | 82 % | NC_003062.2 | 2.84 | 59.4 | 2,765 | 2,819 | 3 |

**Table S2.** COGs analysis of direct plant growth promoting traits

**Table S2.** (continued)

**Table S3.** COGs analysis of indirect plant growth promoting traits

**Table S3.** (continued)

**Table S4.** Some important genes annotated on strain DCY84^T^ genome

| **Name** | **Seq. Description** | **Length** | **#Hits** | **min. eValue** | **Similarity** |
| --- | --- | --- | --- | --- | --- |
| **Proline metabolism and transporter related genes** | | | | | |
| orf00422 | glutamate synthase | 198 | 20 | 1.08E-27 | 85.65 % |
| orf00426 | glutamate synthase | 4308 | 20 | 0 | 86.95 % |
| orf00931 | glutamine synthetase | 1425 | 20 | 0 | 89.65 % |
| orf02576 | glutamate synthase | 1488 | 20 | 0 | 87.70 % |
| orf02637 | glutamine synthetase | 1329 | 20 | 0 | 95.10 % |
| orf06685 | glutamate synthase | 1341 | 20 | 0 | 68.15 % |
| **Polyamine metabolism and transporter related genes** | | | | | |
| orf01990 | spermidine synthase | 744 | 20 | 1.11E-90 | 71.75 % |
| orf02663 | spermidine putrescine abc transporter atp-binding protein | 786 | 20 | 1.04E-128 | 84.25 % |
| orf02808 | spermidine putrescine abc transporter substrate-binding protein | 1092 | 20 | 0 | 80.30 % |
| orf02811 | spermidine putrescine abc transporter permease | 822 | 20 | 4.50E-144 | 83.70 % |
| orf02812 | spermidine putrescine abc transporter atp-binding protein | 789 | 20 | 1.72E-131 | 84.80 % |
| orf02813 | spermidine putrescine abc transporter atp-binding protein | 1065 | 20 | 0 | 85.80 % |
| orf03239 | spermidine putrescine abc transporter atp-binding protein | 144 | 5 | 1.38132 | 50.80 % |
| orf05694 | glutathionylspermidine synthase | 351 | 7 | 1.52063 | 49.86 % |
| orf05702 | spermidine synthase | 930 | 20 | 2.90E-175 | 91.10 % |
| orf06065 | carboxynorspermidine decarboxylase | 1128 | 20 | 0 | 90.75 % |
| orf06278 | spermidine putrescine abc transporter atp-binding protein | 1107 | 20 | 0 | 86.70 % |
| orf06280 | spermidine putrescine abc transporter permease | 804 | 20 | 8.70E-116 | 72.20 % |
| orf06282 | spermidine purescine abc transporter permease | 822 | 20 | 4.50E-130 | 78.30 % |
| orf06284 | spermidine putrescine abc transporter substrate-binding protein | 1074 | 20 | 0 | 90.10 % |

**Table S4.** (continued)

| **Name** | **Sequence Description** | **Length** | **#Hits** | **min. eValue** | **Similarity** |
| --- | --- | --- | --- | --- | --- |
| **Sugar metabolism and transporter related genes** | | | | | |
| orf00379 | beta-glucosidase | 1344 | 20 | 0 | 73.75 % |
| orf00382 | glycoside hydrolase | 1623 | 20 | 3.43E-178 | 64.85 % |
| orf01774 | oligo-glucosidase | 1689 | 20 | 0 | 86.25 % |
| orf02131 | dehydrogenase | 1164 | 20 | 0 | 87.85 % |
| orf02450 | beta-glucosidase | 2229 | 20 | 0 | 78.65 % |
| orf02776 | beta-glucosidase | 2313 | 20 | 0 | 63.45 % |
| orf03226 | alpha amylase catalytic region | 129 | 1 | 1.53136 | 52.00 % |
| orf03686 | alpha-amylase | 2436 | 20 | 0 | 70.55 % |
| orf03697 | alpha-glucosidase | 2319 | 20 | 0 | 80.10 % |
| orf04942 | oligo-glucosidase | 1758 | 20 | 0 | 81.15 % |
| orf04945 | glycosyl transferase family 2 | 1044 | 20 | 6.52E-166 | 70.65 % |
| orf05549 | beta-glucosidase | 2790 | 20 | 0 | 85.10 % |
| orf05549 | beta-glucosidase | 2790 | 20 | 0 | 85.10 % |
| orf06179 | oligo-glucosidase | 609 | 8 | 1.41E-60 | 55.13 % |
| orf06305 | alpha-amylase | 1461 | 20 | 0 | 84.15 % |
| orf06465 | oligo-glucosidase | 1671 | 20 | 0 | 84.10 % |

**Table S4.** (continued)

| **Name** | **Sequence Description** | **Length** | **#Hits** | **min. eValue** | **Similarity** |
| --- | --- | --- | --- | --- | --- |
| **Antibiotic production cluster** | | | | | |
| orf05152 | clp protease | 2442 | 20 | 0 | 93.05 % |
| orf05153 | ATP:guanido phosphotransferase | 1062 | 20 | 0 | 86.30 % |
| orf05154 | activator of protein kinase mcsb | 516 | 20 | 3.27E-94 | 85.15 % |
| orf05155 | transcriptional regulator | 462 | 20 | 1.40E-79 | 83.55 % |
| orf05157 | oxidoreductase | 1254 | 20 | 0 | 70.10 % |
| orf05158 | acetyltransferase | 468 | 20 | 3.03E-82 | 75.70 % |
| orf05161 | NAD-dependent DNA ligase | 2013 | 20 | 0 | 87.05 % |
| orf05164 | ATP-dependent DNA helicase | 2394 | 20 | 0 | 88.85 % |
| orf05165 | heptaprenylglyceryl phosphate synthase | 708 | 20 | 2.20E-110 | 77.25 % |
| orf05171 | UDP -phosphate glucose phosphotransferase | 912 | 20 | 0 | 90.00 % |
| orf05172 | UDP-phosphate glucose phosphotransferase | 495 | 20 | 1.10E-72 | 76.60 % |
| orf05173 | glycosyl transferase family 2 | 960 | 20 | 4.46E-168 | 85.95 % |
| orf05174 | glycosyl transferase | 978 | 20 | 1.93E-106 | 63.60 % |
| orf05175 | dtdp-4-dehydrorhamnose reductase | 774 | 20 | 1.02E-146 | 87.40 % |
| orf05176 | spore coat protein | 1023 | 20 | 0 | 92.25 % |
| orf05177 | dtdp-4-dehydrorhamnose -epimerase | 549 | 20 | 4.21E-118 | 92.60 % |
| orf05178 | spore coat protein | 744 | 20 | 6.83E-156 | 92.10 % |
| orf05181 | polymerase | 1275 | 20 | 0 | 74.90 % |
| orf05182 | DNA-binding protein | 1392 | 20 | 2.75E-143 | 68.65 % |
| orf05183 | peptidase u32 | 2508 | 20 | 0 | 94.45 % |
| orf05185 | deoxycytidine kinase | 675 | 20 | 9.44E-137 | 90.60 % |
| orf05187 | deoxyguanosine kinase | 621 | 20 | 2.20E-115 | 85.10 % |
| orf05189 | copper amine oxidase | 2070 | 20 | 4.85E-117 | 49.85 % |
| orf05190 | trypsin | 1095 | 20 | 6.14E-129 | 72.10 % |
