# Supplementary figures and images for: Complete genome sequence of Paenibacillus yonginensis DCY84T, a novel plant Symbiont that promotes growth via induced systemic resistance
Source: Stand Genomic Sci. 2017 Oct 13;12:63. doi: 10.1186/s40793-017-0277-8 (PMC5640943; doi:10.1186/s40793-017-0277-8)

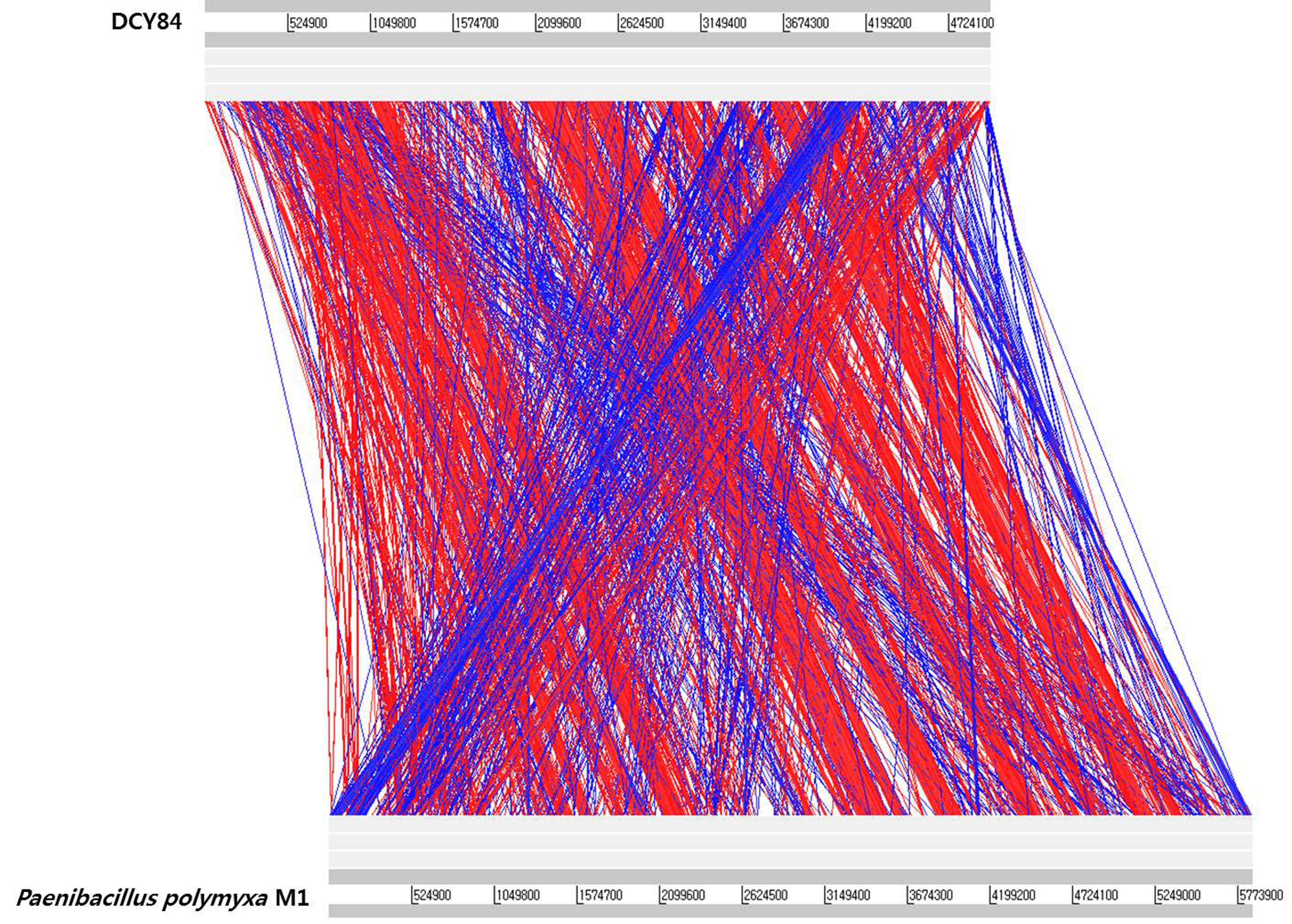

Supplement: Supplementary file 3 — Comparative genome analysis of P. yonginensis DCY84T and P. polymyxa M1 using the Artemis software and ACT. (TIFF 16717 kb) [file 40793_2017_277_MOESM3_ESM.tif]
